# Pregnancy and Poikiloderma with Neutropenia

**DOI:** 10.1055/a-2764-3829

**Published:** 2026-01-22

**Authors:** Jessica Nelson, Hailey Cox, Ira Hamilton, Nauman Khurshid, Nikolina Docheva

**Affiliations:** 1 Department of Obstetrics and Gynecology, University of Toledo and Toledo ProMedica Hospital, Toledo, Ohio, United States; 2 Department of Maternal-Fetal Medicine, Toledo ProMedica Hospital, Toledo, Ohio, United States

**Keywords:** poikiloderma and neutropenia, poikiloderma and pregnancy, chronic neutropenia, congenital neutropenia, neutropenia and pregnancy

## Abstract

**Background:**

Poikiloderma with neutropenia is an autosomal recessive condition characterized by postinflammatory poikiloderma and chronic neutropenia. To date, there is no published literature reporting on the impact of pregnancy on this rare genetic disorder.

**Case:**

This case highlights a successful pregnancy outcome in a 26-year-old gravida 1 para 0 female with poikiloderma with neutropenia. Her pregnancy was complicated by thrombocytopenia, low fetal fraction, and high-risk cell-free DNA result.

**Discussion:**

The findings of low fetal fraction and high-risk aneuploidy were unexpected given normal neonatal outcome. It is not yet well understood whether these findings are related to the genetic condition itself or use of medications used to manage her condition.

## Introduction

Poikiloderma with neutropenia is a rare autosomal recessive condition that primarily affects the integumentary and immune systems. Approximately 100 cases have been described in the literature. 1 It is caused by mutations in the *USB1* gene, located on chromosome 16. This gene encodes for an RNA exonuclease that processes U6 RNA, a component of the spliceosomes, which are essential for pre-mRNA splicing and gene expression regulation. 2 Disruption of this pathway results in damage to U6 RNA due to abnormal splicing. The exact link between USB1 mutations and poikiloderma with neutropenia remains unclear. This condition typically presents as a rash that manifests between 6 and 12 months of age, followed by postinflammatory poikiloderma after 2 years. 1 Chronic neutropenia leads to recurrent sinopulmonary infections, often resulting in bronchiectasis. There is also an increased risk for malignancies such as skin cancer and acute myelogenous leukemia. Characteristic clinical findings include but not limited to; thickened nails, hyperkeratosis on the hands and soles, nonhealing skin ulcers, and calcinosis cutis. Additional features may include short stature, hypogonadotropic hypogonadism, and hepatosplenomegaly. 3

## Case Report

This case details a 26-year-old White female, gravida 1 para 0, who presented to Maternal–Fetal Medicine at 14 ^6/7^ weeks of gestation for consultation regarding her known diagnosis of poikiloderma with neutropenia. This was a spontaneously conceived pregnancy.

Her clinical history revealed a rash at 4 months of age, followed by multiple hospitalizations for infections by age 1. Initially, the patient was suspected to have Rothmund–Thompson syndrome. Her clinical and laboratory findings include but are not limited to: poikiloderma, skin inflammation and atrophy, absent eyelashes and eyebrows, short stature, small vessel vasculitis, calcinosis cutis, immunodeficiency and hypogammaglobulinemia, Hashimoto's thyroiditis, poor dentition, joint contractures, and foot deformities. She underwent genetic testing at age 7, which revealed two mutations in the *USB1* gene suspected to be in trans at c.415C > T and c.673C > T, confirming poikiloderma with neutropenia.

## Pregnancy Course

Her pregnancy was managed in a multidisciplinary fashion including Maternal–Fetal Medicine, Obstetrics, Immunology, Rheumatology, Endocrinology, Hematology, Anesthesia, and Podiatry. The immunology and hematology team managed her chronic neutropenia and hypogammaglobulinemia with daily filgrastim, weekly immunoglobulin subcutaneous (human), and regular monitoring of her immunoglobulins. She continued levothyroxine for Hashimoto's thyroiditis under endocrinology supervision and hydroxychloroquine 200 mg daily for joint pain under rheumatology. She also followed closely with podiatry due to her joint and foot-related symptoms. Pregnancy-specific care included a negative maternal serum α-fetoprotein screen. Cell-free DNA testing at 22 ^6/7^ weeks' gestation revealed a low fetal fraction of 2.2% due to insufficient fetal DNA in sample and high risk for triploidy, trisomy 18, or trisomy 13. She underwent a genetic consultation and declined further testing, including amniocentesis or repeating cell-free DNA. The biological father tested negative for USB1 mutations meaning that the fetus would likely be an obligate carrier based on the fact that the patient carries mutations in both copies of her *USB1* gene. A detailed anatomy scan at 19 weeks was unremarkable except for a low-lying placenta. Serial ultrasounds every 4 weeks confirmed appropriate growth intervals. The patient was also initiated on low-dose aspirin for preeclampsia prevention. Echocardiogram findings were normal despite a history of small vessel vasculitis. She had an abnormal 1-hour glucose tolerance test followed by a normal 3-hour glucose tolerance test. She required prednisone for an asthma exacerbation. Thrombocytopenia developed that was monitored with serial platelet checks. By 32 weeks, the low-lying placenta had resolved. Antenatal fetal surveillance was initiated at 32 weeks with twice-weekly nonstress tests and weekly deep vertical pocket measurements. At 37 ^6/7^ weeks, she developed elevated blood pressures and met criteria for gestational hypertension, prompting medical induction of labor. Routine admission laboratories revealed platelets of 83,000/µL. During her induction she received a cook balloon and oxytocin. The patient was able to receive an epidural for pain control. Special precautions were taken during labor to avoid skin trauma, including gentle positioning and minimizing excessive joint strain. In the event of a cesarean delivery, it was recommended to use gentle tissue handling and avoid adhesive tapes to prevent skin breakdown, She had an uncomplicated vaginal delivery with a second-degree perineal laceration. She delivered a viable male infant, weighing 2,680 g with Apgar scores of 8 and 9. Her postpartum course was complicated by worsening thrombocytopenia with platelets downtrending from 83,000/µL to 58,000/µL. She was discharged home on postpartum day number 2 without any bleeding complications. Her platelets were checked 3 weeks later and had improved to 186,000/µL. Placenta pathology revealed a third trimester placenta, three vessel umbilical cord, marginal cord insertion, and Tenney–Parker changes.

The neonate showed no dysmorphic features or signs of chromosomal abnormalities; therefore, any genetic testing was deferred. He was admitted to the neonatal intensive care unit for 5 days due to hypoglycemia, likely related to maternal steroid use for her skin rash and asthma exacerbations. The patient was discharged on postpartum day 2 without complications.

## Discussion

The patient was continued on daily filgrastim and weekly immunoglobulin subcutaneous (human). There are limited data on the safety of these medications in pregnancy. Filgrastim is a granulocyte colony-stimulating factor and is typically used in oncology settings. Available data thus far have not demonstrated adverse maternal or neonatal outcomes when used in pregnancy. 4 Similarly, immunoglobulin subcutaneous (human) has limited data in pregnancy. Current data do not support a direct link between filgrastim or immunoglobulin subcutaneous (human) with hypertensive disorders of pregnancy. However, a rare side effect of filgrastim can include elevated blood pressures. 5

The abnormal cell-free DNA results demonstrating low fetal fraction and high-risk aneuploidy were unexpected given a normal anatomic ultrasound and neonatal outcome. Maternal autoimmune diseases have been associated with low fetal fractions in some cases. 6 It is also unclear if filgrastim and immunoglobulin subcutaneous (human) played a role in abnormal cell-free DNA results. Currently, there is no evidence that either of these medications are linked to changes in cell-free DNA results, though more studies are needed to explore this association.

## Conclusion

This case highlights a successful pregnancy outcome in a patient with poikiloderma with neutropenia. A multidisciplinary team approach is critical in managing complex comorbidities associated with this condition and in pregnancy. It remains unclear whether the low fetal fraction and high-risk cell-free DNA results were related to her genetic condition or use of medications such as immunoglobulin subcutaneous (human) and filgrastim. Further studies are needed to better understand pregnancy outcomes in patients with this rare condition.

## References

[1] Chekr J Andraws J Elias J Alasmar D Poikiloderma with neutropenia: a case report J Med Case Rep 2025 19 01 16 39810212 10.1186/s13256-025-05027-2 PMC11734469

[2] Didychuk A L Montemayor E J Carrocci T J Usb1 controls U6 snRNP assembly through evolutionarily divergent cyclic phosphodiesterase activities Nat Commun 2017 8 01 497 28887445 10.1038/s41467-017-00484-w PMC5591277

[3] Piccolo V Russo T Di Pinto D Poikiloderma with neutropenia and mastocytosis: a case report and a review of dermatological signs Front Med (Lausanne) 2021 8 680363 34179048 10.3389/fmed.2021.680363 PMC8222900

[4] Berends C Maggen C Lok C AR Maternal and neonatal outcome after the use of G-CSF for cancer treatment during pregnancy Cancers (Basel) 2021 13 06 1214 33802196 10.3390/cancers13061214 PMC8001066

[5] U.S. Food and Drug Administration. Neupogen (filgrastim) [package insert]. Silver Spring (MD): FDA. Accessed June 24, 2025 at:https://www.accessdata.fda.gov

[6] MacKinnon H J Kolarova T R Katz R The impact of maternal autoimmune disease on cell-free DNA test characteristics Am J Obstet Gynecol MFM 2021 3 06 100466 34418590 10.1016/j.ajogmf.2021.100466 PMC8919896

